# Risk stratification of cutaneous melanoma reveals carcinogen metabolism enrichment and immune inhibition in high-risk patients

**DOI:** 10.18632/aging.103734

**Published:** 2020-08-28

**Authors:** Xia Li, Yunpeng Cai

**Affiliations:** 1 Research Center for Biomedical Information Technology, Shenzhen Institutes of Advanced Technology, Chinese Academy of Sciences, Shenzhen 518055, P.R. China

**Keywords:** cutaneous melanoma, biomarker, driver gene, metabolism, immune cell type

## Abstract

Cutaneous melanoma (CM) is the most lethal form of skin cancer. Risk assessment should facilitate stratified surveillance and guide treatment selection. Here, based on the mRNA-seq data from 419 CM patients in the Cancer Genome Atlas (TCGA), we developed a prognostic 21-gene signature to distinguish the outcomes of high- and low-risk patients, which was further validated in two external cohorts. The signature achieved a higher C-index as compared with other known biomarkers and clinical characteristics in both the TCGA and validation cohorts. Notably, in high-risk patients the expression levels of three driver genes, *BRAF*, *NRAS*, and *NF1* in the MAPK pathway, were lower but exhibited a stronger positive correlation as compared with low-risk patients. Moreover, the genes involved in nicotinamide adenine dinucleotide metabolism were negatively correlated with the expression of *BRAF* in the high-risk group. Function analysis revealed that the upregulated genes in the high-risk group were enriched in the cytochrome P450-mediated metabolism of chemical carcinogens. Furthermore, the low-risk group had high levels of gamma delta T cells infiltration, while regulatory T cells were accumulated in the high-risk group. The present study offers a promising new prognostic signature for CM, and provides insight into the mechanisms of melanoma progression.

## INTRODUCTION

Cutaneous melanoma (CM), the most common type of melanoma, is a lethal form of skin cancer. Although it comprises only 3−5% of all skin cancers, it contributes to approximately 75% of all skin cancer-related deaths [1, 2]. Over the last 20 years, the incidence of CM has increased by almost 50% in the United States, from 15/100,000 individuals/year to 22.8/100,000/year [3]. The prognosis of CM patients remains very poor. By the time the tumor has metastasized to the lymph nodes or distant tissues, the 5-year survival rate is only 15–20% [4, 5]. The 10-year survival rate may be as low as 40% when the disease becomes increasingly penetrative of the skin and/or develops local ulceration [6]. Due to its high potential for rapid progression and metastasis [7], risk assessment in CM patients could lead to earlier identification of high-risk disease. It could provide useful information to evaluate prognosis and facilitate appropriate surveillance for the prevention of recurrence or metastasis. Alternatively, identifying patients with low-risk disease would help to avoid unnecessary treatment costs and reduce anxiety.

Clinicopathological features, such as Breslow thickness (mm), ulceration, and microsatellite metastasis, have been traditionally applied to assess and determine overall risk. However, simple classification of tumors based on phenotypic features does not always represent the intrinsic biology of individual tumors, and is limited in its ability to provide an accurate prediction of individual tumor prognosis [8, 9]. This has led to an interest in the identification of molecular biomarkers that can offer alternative tumor risk stratification, in addition to providing insight into complex tumor cell biology.

Gene expression profiling represents a standard preservation approach for purifying RNA from tumor tissue. It has advanced into the clinical setting to provide a robust and reproducible platform for the simultaneous evaluation of a large number of genes. In recent years, a variety of genes have been proposed as prognostic markers in different types of cancer, such as liver, ovarian, and melanoma [10–13]. A previous study in 2014 identified a panel of genes for use as a gene expression signature to define high- and low-risk groups of melanoma patients [12]. RNA from a total of 40 primary Stage II−III melanomas were analyzed to evaluate the expression of 446 immune- or melanoma-related genes, finally obtaining a 53-immune-related gene signature. The signature was validated in a cohort of 48 Stage II−III melanomas, but further validation studies in other populations have not yet been published. Another gene signature of 31 genes was reported as a prognostic marker in 2015 [13]. The prognostic genes were selected from a comparative review of several previous microarray studies, including genes that were associated with metastasis or exhibited expression differences in primary tumors compared with metastatic tumors. The risk for patients was predicted in 164 melanoma tumors, and the ability to segregate risk has since been assessed in additional retrospective and prospective studies [14, 15]. Although it has important clinical use, additional validation in larger populations could allow better performance of the signature.

However, there exist two major limitations regarding the use of these biomarkers in determining the risk for CM. Firstly, as described above, these biomarkers were obtained using a small sample size with a lack of sufficient validation. Subsequent validation in a greater number of patients should ensure reproducibility and reliability. The second limitation lies in the gene set in which the final prognostic genes are distilled. The current signature was determined from a specific gene list, such as immune- or metastasis-related genes. Of note, biomarkers used for the diagnosis of other cancer types were also obtained in a similar way [10, 11], such as based on the differentially expressed genes between tumor and control samples. An important concern in these approaches is missing other new biomarker candidates from the whole transcriptome. Furthermore, genes with differences in expression between tumor and control groups, or metastatic and primary tumors, may also exhibit notable expression differences among tumor samples or metastatic patients. Thus, selection solely from differentially expressed genes may lack consideration of interpatient heterogeneities, and consequently fail to provide adequate prognostic information regarding patient outcomes. To date, few biomarkers provide sufficient prognostic value; therefore, the discovery of new markers for the evaluation of patient prognosis is paramount. In addition, the molecular mechanism contributing to an increased risk in CM patients remains poorly understood. Exploration of the molecular differences between risk groups should advance our knowledge of the underlying risk factors. Precise risk stratification and targeting of high-risk factors could be of great benefit to the prevention and treatment of CM.

In the present study, based on the whole transcriptome profiles of 419 patients with CM from the Cancer Genome Atlas (TCGA), we developed a new prognostic signature that successfully classified patients into high- and low-risk groups with significantly different outcomes. Further analysis confirmed that the signature had superior ability in predicting the overall survival (OS) of patients than other known biomarkers and clinical characteristics. The prognostic value of the signature was further validated in two external cohorts. Moreover, we observed a different expression pattern of the driver genes in the two risk groups and the major functional differences existed in the enrichment of metabolic pathways and levels of infiltration of different immune cell types.

## RESULTS

### Identification of high- and low-risk patients using the 21-gene signature

**Figure 1:**
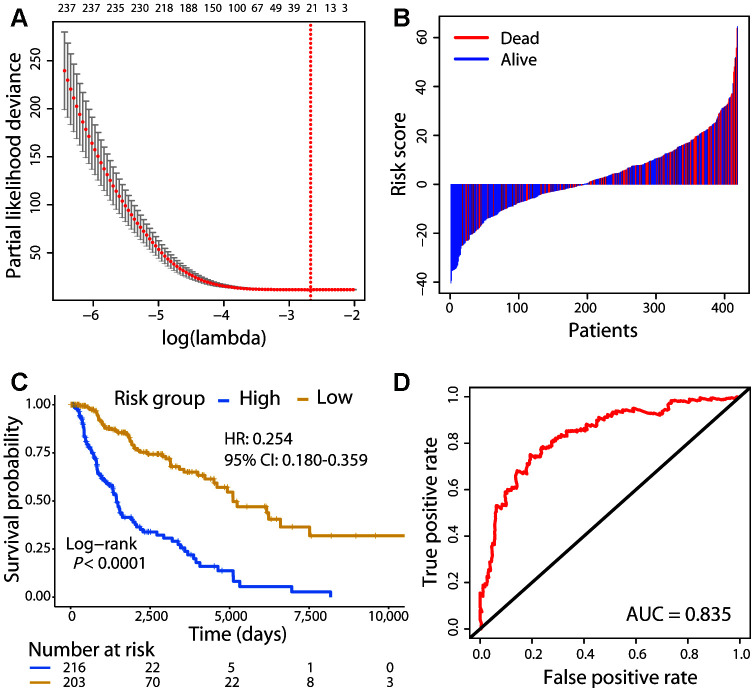
**Risk stratification of CM patients using the 21-gene signature.** (**A**) Cross-validation error plot for tuning parameter selection in the proportional hazards model. The R package glmnet returns a sequence of lambda values and cross-validation chooses the optimal value. The plot includes the cross-validation curve (red dotted line) and upper and lower standard deviation (error bars) along the lambda sequence. We used the lambda value with a minimum standard error of the mean (red vertical line) across 100 runs to choose the 21-gene signature. (**B**) Risk score distribution with patient survival status. The *x* axis is sorted by risk score values. Red indicates dead patients and blue indicates those still alive. (**C**) The Kaplan–Meier survival analysis of the signature. Patients were divided into high- and low-risk groups using the cut-off value estimated by the R package survminer. (**D**) ROC analysis of the signature in predicting the OS of patients.

A total of 419 patients with CM, for whom both transcriptome data and clinical information were available, were included in the present study. Among 19,620 protein-coding genes, 1,086 were associated with OS (Log-rank test, *P* < 0.01) according to univariate Cox regression analysis. A lasso-penalized Cox proportional hazards model was applied to choose a gene set with the best prognostic value (Figure 1A). Consequently, a new signature of 21 genes was identified and selected, including *SATB1*, *HN1L*, *CCL8*, *TTC39C*, *HPDL*, *OLFML2A*, *LMNTD2*, *ATP11A*, *SLC5A3*, *HEYL*, *BOK*, *RBCK1*, *CCT6B*, *ABTB1*, *CLEC18A*, *MRPS6*, *NXT2*, *SPEF2*, *KLK13*, *SPAG8*, and *COL22A1* (Supplementary Table 1). None of these 21 genes have been reported as CM biomarkers in previous studies.

A risk score for each patient was calculated using the expression value of the 21 genes and their regression coefficients from Cox analysis, similar to a previously reported approach [16]. The risk score distribution with survival status was shown in Figure 1B. Patients were divided into high-risk (n = 216) and low-risk (n = 203) groups using the cut-off value calculated by the survminer R package. Survival analysis found that patients in the high-risk group had a significantly shorter OS than those in the low-risk group (Hazard ratio [HR]: 0.254, 95% confidence interval [CI]: 0.180−0.359; Log-rank test, *P* < 0.01; Figure 1C). The concordance statistic (C-index) was used to evaluate the predictive accuracy; the C-index of the 21-gene signature for the prediction of OS was 0.679. Moreover, receiver operating characteristic (ROC) analysis was also performed, and the AUC (area under the ROC curve) for the 5-year OS was 0.835 (Figure 1D). These results indicated that our prognostic gene model achieved good performance in distinguishing between high- and low-risk patients.

### Comparison of the 21-gene signature with other known prognostic biomarkers

**Figure 2:**
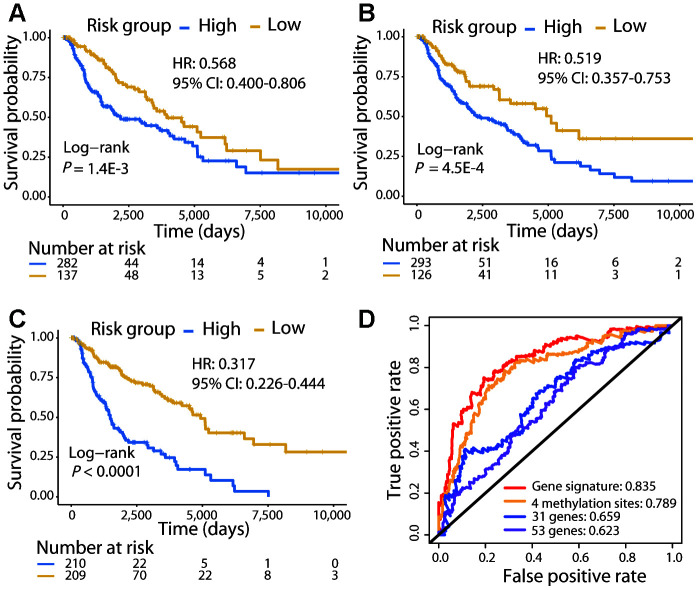
**Comparison of the 21-gene signature with known biomarkers in predicting the OS in the TCGA cohort.** Kaplan–Meier analysis was performed for patients classified by the 31-gene (**A**), 53-gene (**B**), and methylation (**C**) signatures. (**D**) ROC curves of the three known signatures and the 21-gene signature are shown. The AUC values of each signature demonstrate their ability to predict the patient OS.

We compared the patient survival prediction ability of the 21-gene signature with other known biomarkers, 31-gene [13] and 53-gene [12] signatures identified in previous studies. Moreover, a recent study reported methylation at 4 specific sites as a prognostic biomarker for CM, demonstrating superior ability to predict the OS of patients than numerous known prognostic markers [17]; thus, this methylation signature was also included in our comparison. The 419 CM patients were divided into high- and low-risk groups using each signature, and survival analysis showed that patients in the high-risk group were significantly associated with a poorer OS (Log-rank test, *P* < 0.01; Figure 2A−2C). The C-index of the 31-gene, 53-gene, and methylation signatures were 0.595, 0.573, and 0.638, respectively, each of which was lower than that of the 21-gene signature (C-index: 0.679). Furthermore, the AUC for the 21-gene signature was much higher than that for the other three signatures (Figure 2D). These results demonstrated the powerful ability of the 21-gene signature to predict prognosis and outperform the other signatures.

### External validation of the risk prediction ability of the 21-gene signature

**Figure 3:**
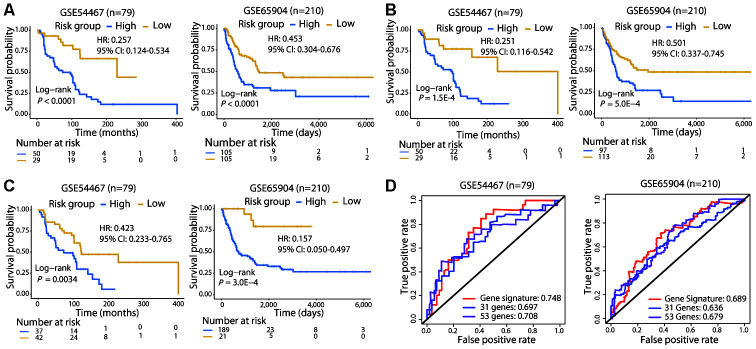
**Survival and ROC curves of the different signatures in the validation cohorts.** Survival analysis of patients classified by the 21-gene (**A**), 31-gene (**B**), and 53-gene (**C**) signatures in the two validation cohorts. (**D**) ROC curves and AUC values of the three signatures in the two validation cohorts. In GSE65904, the AUC value for 3-year OS was calculated, since few patients lived for 5 years.

To validate the 21-gene signature in other populations, we calculated the risk score for melanoma patients in GSE54467 (n = 79) and GSE65904 (n = 210) using the same formula and performed risk stratification in the same way as with the TCGA cohort. Consistent with the results of the TCGA cohort, patients in the high-risk group exhibited a significantly poorer OS than those in the low-risk group in both validation populations (Log-rank test, *P* < 0.01; Figure 3A). In addition, we used the 31-gene and 53-gene signatures to separate patients into high- and low-risk groups in these two validation cohorts. Both of these signatures successfully categorized patients, and the survival curves showed that the low-risk group had a longer OS than the high-risk group (Log-rank test, *P* < 0.01; Figure 3B, 3C).

Subsequently, we compared the prognostic value of the 21-gene signature with the two signatures in the two validation cohorts. The C-index of the 21-gene signature had a higher value (GSE54467: 0.644; GSE65904: 0.616) compared with that of the 31-gene (GSE54467: 0.626; GSE65904: 0.573) and 53-gene (GSE54467: 0.599; GSE65904: 0.565) signatures in the validation cohorts. Moreover, the 21-gene signature also achieved a higher AUCs than the two signatures in GSE54467 and GSE65904 (Figure 3D). Collectively, the prognostic capacity of the 21-gene signature was not only good in the TCGA cohort but also in the validation dataset, exhibiting superior ability to the two other known signatures.

### Comparison of risk prediction with other clinical factors

We further performed univariate and multivariate Cox regression analyses to evaluate the prognostic independence of the 21-gene signature. Clinical features including gender, age, Breslow thickness, ulceration, pathological stage, tumor site, and metastasis were compared with the 21-gene signature. The results showed that the 21-gene signature (risk score) and pathological stage were both significantly correlated with patient OS, independent of other factors (Table 1). However, the HR of the risk score indicated a 74.6% reduction in the risk of death in the low-risk group, but only a 46.8% reduction in the early stage (Stages 0, I, and II) group. The C-index of the pathological stage was 0.594, which was also lower than that of the 21-gene signature (0.679). In addition, we also compared their performance in the two validation datasets (Supplementary Table 2). In GSE54467, only the risk score was associated with OS (Log-rank test, *P* < 0.01). Both the risk score and tumor stage were significantly correlated with prognosis in GSE65904; however, only the risk score was significantly associated with OS in the multivariate analysis (Log-rank test, *P* < 0.01). Again, the 21-gene signature had a higher C-index (0.616) than that of the tumor stage (0.61) in GSE65904. These results demonstrated better risk prediction of the 21-gene signature than currently used clinicopathological prognostic factors.

**Table 1:** Comparison of the risk prediction ability between the 21-gene signature and clinical factors.

**Variable**	**Group**	**Univariate**		**Multivariate**	
		**HR (95% CI)**	***P* value**	**HR (95% CI)**	***P* value**
Risk score	High (n = 216)	1	**1.11E-16**	1	
	Low (n = 203)	0.254 (0.180-0.359)		0.253 (0.152-0.422)	**1.36E-7**
Gender	Male (n = 264)	1.129 (0.796-1.601)	0.496		
	Female (n = 155)	1			
Breslow thickness (mm)	≤ 1 (n = 53)	1		1	
	1.01-2 (n = 77)	0.925 (0.529-1.619)	**3.75E-6**	0.552 (0.259-1.177)	0.124
	2.01-4 (n = 67)	1.558 (0.886-2.739)		0.550 (0.253-1.195)	0.131
	≥ 4 (n = 122)	2.962 (1.735-5.056)		0.724 (0.326-1.609)	0.428
Ulceration	Yes (n = 146)	1.953 (1.290-2.955)	**0.001**	1.605 (0.952-2.704)	0.076
	No (n = 132)	1		1	
Age	≤ 60 (n = 233)	0.575 (0.410-0.807)	**0.001**	0.590 (0.371-0.939)	0.026
	> 60 (n = 186)	1		1	
Pathological stage	Stage 0, I, and II (n = 203)	1	**4.84E-4**	1	
	Stage III and IV (n = 180)	1.880 (1.312-2.694)		2.326 (1.411-3.836)	**9.36E-4**
Tumor site	Extremities (n = 181)	1	0.111		
	Head and neck (n = 31)	1.451 (0.784-2.685)			
	Trunk (n = 145)	0.847 (0.586-1.225)			
	Other (n = 13)	1.921 (0.878-4.200)			
Metastasis	Primary tumor (n = 78)	4.657 (1.538-4.657)	**8.51E-6**	1.953 (0.682-5.590)	0.212
	Regional lymph node (n = 208)	1.022 (0.678-1.539)		0.895 (0.511-1.567)	0.698
	Regional cutaneous or subcutaneous tissue (n = 70)	0.784 (0.457-1.343)		0.483 (0.228-1.021)	0.057
	Distant metastasis (n = 60)	1		1	

### Strong correlation of *BRAF*-*NRAS*-*NF1* expression in the high-risk group and weak correlation in the low-risk group

**Figure 4:**
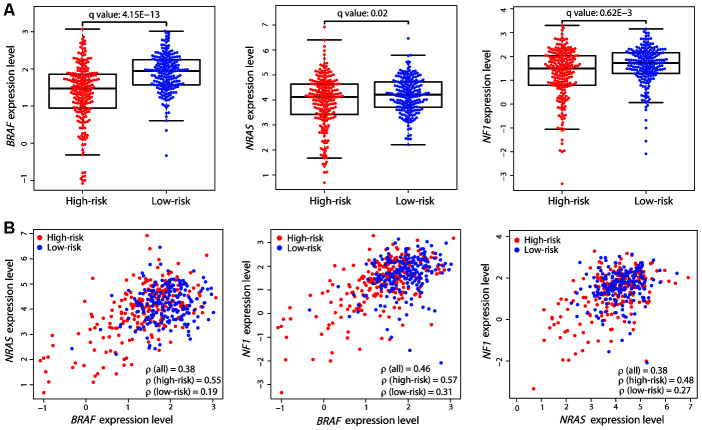
**Expression differences and correlation of the three driver genes between the two risk groups.** (**A**) Distribution of the expression levels of *BRAF*, *NRAS*, and *NF1* in the two risk groups. The expression levels are represented by log2-transformed RPKM values. The *P* value was calculated by a two-sided Student’s *t* test and adjusted using the Benjamini−Hochberg method (*q* value). (**B**) Correlation between the expression levels of the three driver genes. Each dot represents one patient (high-risk, red dot; low-risk, blue dot). The correlation coefficient ρ was calculated by Spearman’s rank analysis. Coefficient values between two genes among all patients, high-risk patients, and low-risk patients are shown.

The above results demonstrated the robust performance of the 21-gene signature in prognosis prediction. Known driver genes, including *BRAF*, *NRAS*, and *NF1*, have been previously identified for CM; therefore, we compared mutation differences between the two risk groups using the exome-sequencing data of the same patients. Only *BRAF* showed a significantly high mutation load in the low-risk group (89/215 vs. 114/202, Fisher’s exact test, *P* = 2.389E-3). Gene expression comparison revealed that the expression level of *BRAF* was also significantly higher in low-risk patients (Student’s *t* test, adjusted *P* value = 4.15E-13; Figure 4A). The other two driver genes, *NRAS* and *NF1*, also had higher expression levels in the low-risk group. We calculated the Spearman’s rank correlation coefficient (ρ) between the expression levels of the driver genes in all patients and found good correlation (Figure 4B). However, interestingly, there was a strong correlation in the high-risk group but a weak correlation in the low-risk group, despite these genes being upregulated in the latter (Figure 4B). The strong correlation of the expression levels of these driver genes in the high-risk group may be associated with poor prognosis in these patients.

### The genes involved in nicotinamide adenine dinucleotide (NAD) metabolism exhibited a negative correlation with *BRAF* expression

**Figure 5:**
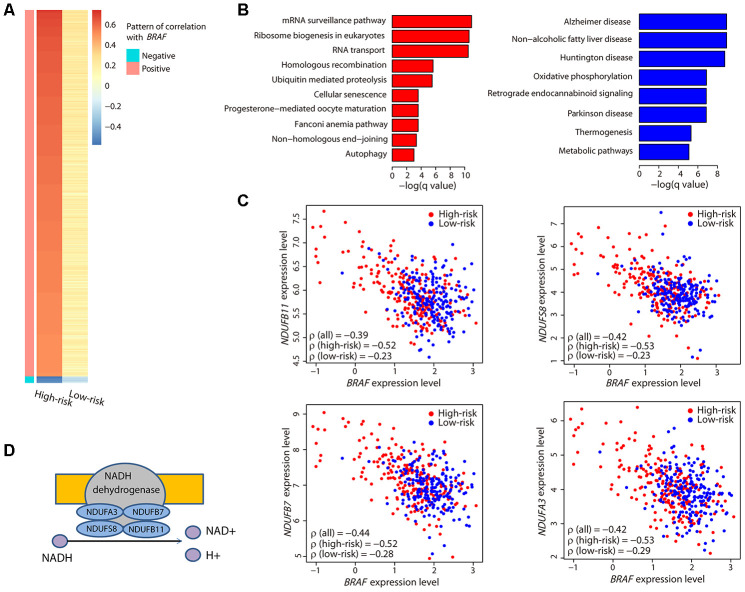
**Characterization of the *BRAF*-correlated genes.** (**A**) The heatmap illustrates all ρ values of genes that had a strong correlation with *BRAF*. Only two sets of genes exhibiting a strong positive or negative correlation in the high-risk group and a weak correlation in the low-risk group were obtained. (**B**) Bar plots showing the significantly (adjusted *P* value < 0.05) enriched KEGG pathways related to the positively (red) and negatively (blue) correlated genes. (**C**) Correlation between the expression levels of the four genes encoding the subunits of NADH dehydrogenase and *BRAF*. Coefficient values between two genes among all patients, high-risk patients, and low-risk patients are shown. (**D**) Illustration showing the four genes encoding the subunits of NADH dehydrogenase and their function.

To obtain other correlation patterns, we further calculated the Spearman’s ρ values between the expression level of *BRAF* and those of all genes in the transcriptome of patients in each risk group. We defined a strong positive correlation as ρ > 0.5, a strong negative correlation as ρ < -0.5, and a weak correlation as -0.3 < ρ < 0.3. When examining all ρ values, there were two sets of genes with correlation patterns: 1) those with a strong positive correlation with *BRAF* in the high-risk group and a weak correlation in the low-risk group; and 2) those with a strong negative correlation with *BRAF* in the high-risk group and a weak correlation in the low-risk group (Figure 5A).

Function analysis revealed that the positively correlated genes were involved in mRNA processing, and the negatively correlated genes were enriched in metabolic pathways (Figure 5B). Specifically, we noticed that four genes, *NDUFA3*, *NDUFB7*, *NDUFS8*, and *NDUFB11*, were present in all the metabolic pathways. Interestingly, these four genes encode the subunits of NADH dehydrogenase that catalyzes the formation of NAD (Figure 5D). Each of these genes exhibited a strong negative correlation with *BRAF* (Figure 5C) and was highly expressed in the high-risk group (Supplementary Figure 1). These data suggested that the NAD metabolic pathway, perhaps in conjunction with *BRAF* expression, may be important in CM patient survival.

### Cytochrome P450-mediated metabolic pathways were highly enriched in the high-risk group

**Figure 6:**
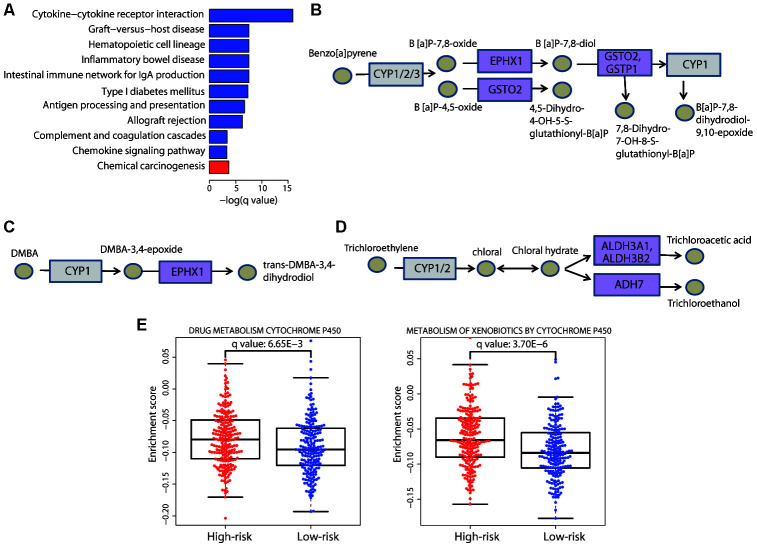
**Enrichment of the cytochrome P450-mediated metabolic pathways in the high-risk group.** (**A**) Bar plot showing the significantly (adjusted *P* value < 0.05) enriched KEGG pathways in the high-risk (red) and low-risk (blue) patients. The chemical carcinogenesis function was significantly enriched in high-risk patients. The specific chemical metabolic pathways are illustrated in (**B**−**D**); in these three pathways, each circle represents one chemical compound and each rectangle represents an enzyme. The cytochrome P450 enzymes are marked in gray and the enzymes with altered gene expression are marked in purple. (**E**) Distribution of the enrichment scores of the cytochrome P450-mediated pathways that showed significant differences (adjusted *P* value < 0.01) between the two risk groups. Each dot represents one patient. The enrichment score of each patient was calculated by ssGSEA. The *P* value was calculated by a two-sided Student’s *t* test and adjusted using the Benjamini−Hochberg method.

To determine the global functional differences, we next investigated the differentially expressed genes between the two risk groups and compared their enriched biological functions. Kyoto Encyclopedia of Genes and Genomes (KEGG) analysis revealed that immune pathways were upregulated in the low-risk group and the chemical carcinogenesis function was significantly enriched in the high-risk group (Figure 6A).

We carefully examined the components that were disordered in the chemical carcinogenesis function and found that the altered signal-transduction required cytochrome P450 enzymes. Specifically, the altered genes were members of three cytochrome P450-mediated metabolic pathways. The first pathway involved the metabolism of the environmental carcinogen benzo[a]pyrene (BaP) (Figure 6B), a cancer-causing agent, exposure to which can increase the risk of skin cancer. Three genes were upregulated in this pathway: *EPHX1*, *GSTO2*, and *GSTP1*. Activation of this pathway converts BaP to B[a]P-7,8-dihydrodiol-9,10-epoxide, a DNA-reactive intermediate. *EPHX1* is also a member of another enriched pathway, the metabolism of DMBA (Figure 6C); DMBA is a widely used chemical compound to induce skin cancer in animal models [18]. Similarly, this pathway activates the metabolism of DMBA via cytochrome P450, resulting in the formation of DNA adducts. These two pathways both contribute to the development of skin cancer, and exposure to these carcinogens is associated with a greater risk of its occurrence. Thus, these results verified the rationale for our risk stratification. Moreover, we noticed that three upregulated genes in the high-risk group, *ALDH3A1*, *ALDH3B2*, and *ADH7*, participate in the cytochrome P450-mediated metabolism of trichloroethylene (Figure 6D); trichloroethylene is an organic chemical, exposure to which can cause cancer. Our result suggested that it may also be associated with an increased risk of CM.

Furthermore, we also performed single sample gene set enrichment analysis (ssGSEA) and the results confirmed the significant enrichment of cytochrome P450-mediated metabolic pathways in the high-risk group (Figure 6E). Collectively, these results demonstrated that the upregulation of cytochrome P450-mediated metabolic pathways had a deleterious effect on CM patient survival, highlighting the necessity to examine patient exposure to chemical carcinogens.

### The high-risk group displayed immune inhibition and the low-risk group was enriched in gamma delta T cells

**Figure 7:**
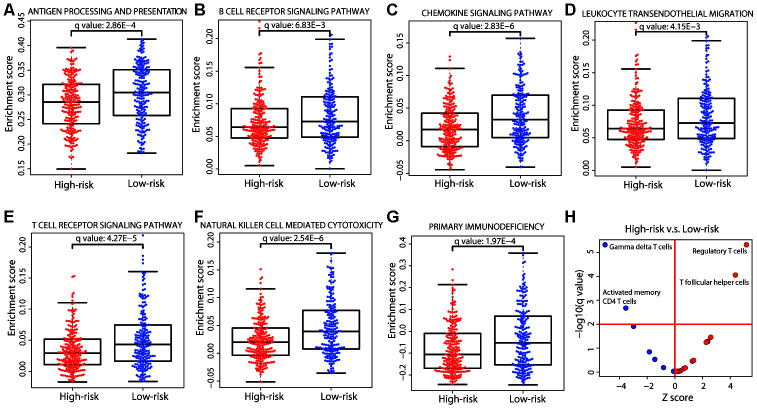
**Immune differences between the two risk groups.** (**A**−**G**) Distribution of the enrichment scores of the immune pathways that were significantly different (adjusted *P* value < 0.01) between the two risk groups. Each dot represents one patient. The enrichment score of each patient was calculated by ssGSEA. The *P* value was calculated by a two-sided Student’s *t* test and adjusted using the Benjamini−Hochberg method. (**H**) Immune cell composition differences between the high- and low-risk groups. The *P* values were calculated by the Wilcoxon rank-sum test and adjusted using the Benjamini−Hochberg method. The red horizontal line represents an adjusted *P* value of 0.01 and the red vertical line indicates a Z score of 0. The analysis was performed for all 22 immune cell types but only those that were significant are labeled on the plot.

Immune system-related genes were upregulated in the low-risk group (Figure 6A); consistently, the immune pathways in the ssGSEA analysis were significantly enriched in the low-risk group (Figure 7A−7G). We further investigated the cellular composition of immune infiltrates by quantitating the fraction of different immune cell types in the two risk groups. The cell fractions of a total of 22 immune cell types were estimated using the CIBERSORT algorithm [19]. The results showed a similar pattern of immune composition of the majority of immune cell types between the high- and low-risk groups (adjusted *P* value > 0.01; Figure 7H). However, in the high-risk group, we observed a depletion of gamma delta and activated memory CD4 T cells, and a significant enrichment of regulatory T (Treg) cells and T follicular helper cells (adjusted *P* value < 0.01). Tregs have immunosuppressive functions in cancer, such as inhibiting recognition and clearance of tumor cells [20]. Gamma delta T cells, defined by the expression of heterodimeric T-cell receptors composed of γ and δ chains, show tissue-specific localization and are enriched in skin and mucosal tissues. Evidence has shown that the infiltration of gamma delta T cells in a tumor was the best predictor of a favorable outcome [21]. The enrichment of gamma delta T cells in the low-risk group was consistent with a better prognosis, further supporting the rationale for our risk prediction. Therefore, our results indicated that immune inhibition existed in the high-risk group and immune activation existed in the low-risk group.

## DISCUSSION

The clinical features of CM have traditionally been used to assess patient risk; however, this method faces limitations since it does not consider the intrinsic heterogeneity of CM. Some patients diagnosed with thin melanomas (Breslow thickness < 1 mm) display variable 10-year survival outcomes ranging from 85% to 99%, and conversely, some patients with thicker melanomas may be cured by surgical management [22]. Molecular stratification of CM, such as gene signatures based on mRNA expression [12, 13], has shown great potential for prognosis prediction and disease management. The development of new biomarkers to precisely assess tumor prognosis would help us to better understand the underlying biology of high-risk patients, leading to improved treatment options.

In the present study, using the transcriptome profiles of 419 CM patients, we developed a new 21-gene prognostic signature with a powerful ability to stratify patients into high- and low-risk groups. The signature showed consistent prognosis prediction in two external validation cohorts and exhibited superior ability to other known signatures and clinicopathological factors. Among the 21 genes, several have been reported as biomarkers for other cancer types or diseases. *ATP11A* and *BOK* are new biomarkers for colorectal cancer [23, 24]. Interestingly, the BCL-2 family protein BOK has been proposed to act in a pro-apoptotic pathway [25]. Links between *BOK* expression and patient outcome have not been reported in melanoma to date. In our data, increased expression of *BOK* was associated with poor OS in CM patients (HR: 2.337, 95% CI: 1.476−3.700; Log-rank test, *P* < 0.01; Supplementary Table 1). When examining the differentially expressed genes between the two risk groups, we found that *BOK* was significantly upregulated in the high-risk group (adjusted *P* value < 0.01). Owing to its role in apoptosis in tumor cells, these findings contradict our expectations. It has been reported that the expression level of *BCL-2* is increased during the development and progression of melanoma [25]. Notably, the function of *BOK* in mammalian cells has not been well characterized and various other functions, such as a role in metabolism, have also been reported [26]. Its function and regulation in melanoma warrant further investigation. The upregulation of *COL22A1* has been proposed as a useful prognostic predictor in patients with squamous cell carcinoma of the head and neck [27]. The expression of *CLEC18* has been demonstrated as a potential biomarker in patients with chronic hepatitis B infection [28]. Different expression levels of *KLK13* have been shown to be associated with patient prognosis in esophageal squamous cell carcinoma [29] and bladder cancer [30]. The transcription regulator *HN1L*, identified as a new oncogene, is considered a potential biomarker for non-small cell lung cancer [31, 32]. Our study demonstrates that these genes also serve as a prognostic signature in CM, adding new prognostic value.

Intriguingly, the expression level of the most common oncogene in melanoma, *BRAF*, was significantly lower in the high-risk group as compared with that in the low-risk group. BRAF inhibitors have proven to be highly effective in targeting the oncogenic BRAF protein in melanoma patients [33]. However, the response to BRAF inhibitors varies among patients, as does the expression of *BRAF*, and there is no correlation between its expression and response to BRAF inhibitors or survival [34]. Wilmott et al. suggested that the expression of *BRAF* did not predict the response or survival of patients, and hypothesized that patients with a low expression of *BRAF* would have a reduced survival rate as compared with those with a high expression [34]. This hypothesis is consistent with our observation that the high-risk patients actually harbored a low expression level of *BRAF*. The lack of correlation may be explained by the fact that melanoma survival is determined by complex molecular mechanisms. Our data showed that the expression levels of three driver genes, *BRAF*, *NRAS*, and *NF1*, were strongly positively correlated in the high-risk patients and weakly correlated in the low-risk patients. The interesting correlation pattern indicated that it may be the correlation pattern of each driver gene, not the expression level, that contributes to the high-risk tendency. On the other hand, our study reveals for the first time that the expression of genes involved in the NAD metabolic pathways are negatively correlated with the expression of *BRAF* in high-risk CM patients. Moreover, the functions of the genes that were upregulated in the high-risk group were enriched in cytochrome P450-mediated metabolic pathways. Taken together, these findings support a connection between metabolic dysregulation and high-risk CM patients.

Metabolic adaptation of cancer cells is required to support proliferation, growth and survival [35]. The present study shows that the expression levels of four genes encoding subunits of NADH dehydrogenase were negatively correlated with the expression of *BRAF* and upregulated in high-risk patients. NADH dehydrogenase catalyzes the oxidation of NADH to NAD, the latter of which is a key cofactor for energy transduction in metabolic processes. Increased levels of NAD result in metabolic alterations in cancer cells [36, 37]. It has been hypothesized that drugs interfering with the NAD biosynthetic enzyme would stop tumor growth [38]. Our results suggest that NADH dehydrogenase represents a new therapeutic target in CM patients. Interestingly, the inhibition of *BRAF* in melanoma cell lines has been reported to achieve high levels of NAD, which were activated by the overexpression of nicotinamide phosphoribosyltransferase (NAMPT), the most important NAD biosynthetic enzyme [39]. These observations are in accordance with our data demonstrating a negative correlation between the expression of *BRAF* and NADH dehydrogenase. *BRAF* may act as an important regulator in the metabolic alterations of CM cells.

Function analysis of the genes that were significantly upregulated in the high-risk group showed enrichment of cytochrome P450-mediated metabolic pathways, which also suggests metabolic alterations in high-risk patients. Many studies have reported the presence of cytochrome P450 enzymes in tumors and their role in the promotion of cancer progression [40, 41]; however, the involvement of cytochrome P450 in melanoma has not been well studied. Our observation that genes involved in this pathway were expressed at higher levels in the high-risk group offer a potential therapeutic option using these enzymes as drug targets. The enrichment of pathways metabolizing substrates of cytochrome P450 enzymes, such as BaP, DMBA, and trichloroethylene, in the present study, suggest that exposure to any toxic substance, such as smoking or drinking contaminated water, should be considered a lifestyle risk factor for screening CM patients in the future.

Melanoma has long been a core focus of ongoing immunotherapy research [42], and several therapeutic strategies have been approved by the FDA for clinical use [43–45]. However, resistance has been reported in a proportion of patients, with studies demonstrating that the existence of multiple immunosuppressive pathways in the tumor microenvironment is intrinsically responsible for failure of immunotherapy [46–48]. Here, we reveal that immunosuppressive Tregs were accumulated in high-risk patients, suggesting that this group may acquire resistance to immunotherapy. Notably, metabolic dysregulation has been reported to occur with T cells, which consequently favors the development of Tregs [49]. Interestingly, the NAD pathway enzymes are receiving increasing attention due to their roles in several aspects of immune cell fate and function [50], once again highlighting that the inhibition of NAD synthesis could restore metabolic balance in the tumor microenvironment. Therefore, approaches inhibiting Treg functions or removing these cells directly from the tumor microenvironment could serve as new immunotherapeutic strategies for CM. On the other hand, our study shows that gamma delta T cells exhibited a high level of infiltration in low-risk patients, suggesting another promising treatment for CM utilizing these cells. In fact, there has been great interest in exploring the therapeutic potential of gamma delta T cells in different types of tumors, given the safety and favorable efficacy displayed in clinical trials [21, 51, 52]. Our study suggests that CM patients may benefit from immunotherapy that increases gamma delta T cells or inhibits Treg functions.

In summary, the present study developed a new 21-gene prognostic signature for CM risk assessment by considering genes from the entire transcriptome and deciphered the underlying mechanisms contributing to risk. The use of this signature may promote further biomarker discovery for the improvement of patient OS, and the uncovered mechanisms can aid the development of new therapeutic targets for CM.

## MATERIALS AND METHODS

### Data source

We collected data regarding 419 patients with skin cutaneous melanoma (SKCM), whose gene expression profile and clinical information were available, from Genomic Data Commons (GDC) (https://portal.gdc.cancer.gov/) (April 12, 2018). Both the normalized gene expression values (RPKM [reads per kilobase per million mapped reads]) and the raw reads counts data of the same patients were downloaded from the mRNA-seq platform (Illumina HiSeq 2000 RNA Sequencing). The log2-transformed RPKM values were used to represent the expression levels. Entrez IDs were used to represent genes, and a total of 19,620 protein-coding genes were retained for downstream analysis. For validation purposes, two sets of gene expression microarrays, GSE54467 (n = 79) and GSE65904 (n = 210), were obtained from the National Center for Biotechnology Information Gene Expression Omnibus (NCBI GEO, https://www.ncbi.nlm.nih.gov/geo/). The corresponding clinical information was also acquired.

### Gene signature selection and patient risk classification

For each gene, the top quartile of patients with higher expression levels were selected as the high expression group and the bottom quartile of patients were selected as the low expression group. Genome-wide survival analysis was performed using the univariate Cox proportional hazards regression model in the survival package of the R platform (version 3.5.1) (R Core Team, Vienna, Austria). Genes with *P* values less than 0.01 were determined as significantly correlated with patient survival.

Subsequently, significant genes were entered into a lasso-penalized Cox proportional hazards model using the R package glmnet to select the optimal prognostic genes. A 10-fold cross-validation and a maximum number of 100,000 iterations were applied. We performed 100 repeat runs to avoid randomization of the results, obtaining a lambda value with the minimum standard error of the mean, which was used to extract the gene signature. The risk score for each patient was calculated as the sum of the expression level of each gene multiplied by its corresponding regression coefficient, as previously reported [16]: $\;\text{risk score}=\sum_{i=1}^{k}\beta_{i}E_{i},$ where *β_i_* was the regression coefficient from the Cox analysis, and *E_i_* was the expression level of the *i*th gene.

Based on the risk score, an optimal cut-off value was estimated by the R package survminer, and the patients were classified into high- and low-risk groups according to the threshold. A survival curve was generated using the Kaplan−Meier method, and differences were evaluated using the Log-rank (Mantel−Cox) test. The HR and corresponding 95% CI were obtained using the Cox proportional hazards model.

The concordance C-index value was applied to evaluate the predictive accuracy. A larger C-index indicated a more accurate predictive ability of the model. In addition, ROC curve analysis was used to evaluate the predictive value of the risk score using the R package survivalROC. The AUC value for 5-year OS was calculated.

For the purpose of comparison with the methylation signature, the methylation data (Illumina Human Methylation 450 platform, beta values) of the same CM patients were downloaded from the GDC (April 12, 2018). Beta values were measured as the ratio of the methylated probe intensity over all methylation probe intensities and were used to represent the relative methylation level. The predictive value of the risk score was investigated in the same manner in both the TCGA cohort and the validation cohorts.

### Comparison of the signature with clinical factors

Clinical parameters including gender, Breslow thickness, ulceration, age, pathological stage, tumor site, and metastasis were included for comparison and independent investigation. Patients were divided into different groups according to clinical characteristics: gender (male, female), Breslow thickness (≤ 1 mm, 1.01−2 mm, 2.01−4 mm, and ≥ 4 mm), ulceration (yes, no), age (≤ 60, > 60), pathological stage (early stage [Stage 0, I, and II], later stage [Stage III and IV]), tumor site (extremities, head and neck, trunk, and other), and metastasis (primary tumor, regional lymph node, regional cutaneous or subcutaneous tissue, and distant metastasis). Univariate and multivariate analyses were performed using the Cox regression model. The validation datasets followed the same grouping and analysis procedure.

### Analysis of driver genes in both risk groups

We downloaded the exome-sequencing data of the same SKCM patients from GDC (April 12, 2018) and obtained the nonsynonymous mutations. For comparison of the gene mutation burden between the two risk groups, we calculated the number of patients in each group harboring the mutated gene, and Fisher’s exact test was applied to evaluate the difference. A *P* value less than 0.01 was considered significant.

The differences in the gene expression levels between the two groups were evaluated by two-sided Student’s *t* tests. The *P* value was adjusted using the Benjamini−Hochberg method. Spearman’s rank analysis was performed to assess the correlation of gene expression with the whole cohort and with the high- and low-risk groups.

### Correlation analysis of the expression of genes in the entire transcriptome with that of *BRAF*

Based on genes in the entire transcriptome, we performed Spearman’s rank analysis to assess the association of their expression levels with that of *BRAF* in each risk group. We defined three categories of correlation: a strong positive correlation as ρ > 0.5, a strong negative correlation as ρ < -0.5, and a weak correlation as -0.3 < ρ < 0.3. We then extracted genes that fell into six correlation patterns: 1) a strong positive correlation in the high-risk group and a weak correlation in the low-risk group; 2) a strong positive correlation in the high-risk group and a strong negative correlation in the low-risk group; 3) a weak correlation in the high-risk group and a strong positive correlation in the low-risk group; 4) a weak correlation in the high-risk group and a strong negative correlation in the low-risk group; 5) a strong negative correlation in the high-risk group and a strong positive correlation in the low-risk group; and 6) a strong negative correlation in the high-risk group and a weak correlation in the low-risk group. Finally, we obtained two sets of genes: 1) those with a strong positive correlation with *BRAF* in the high-risk group and a weak correlation in the low-risk group; and 2) those with a strong negative correlation with *BRAF* in the high-risk group and a weak correlation in the low-risk group. Function analysis of these two sets of genes was performed using WebGestalt [53]. The *P* value was adjusted using the Benjamini−Hochberg method. A pathway was considered significantly enriched at an adjusted *P* value less than 0.05.

### Analysis of gene expression and functional differences between the two risk groups

We applied the R package DESeq [54] using the mRNA-Seq raw reads count data to identify the differentially expressed genes. The *P* value was adjusted using the Benjamini−Hochberg method. We defined genes as differentially expressed at an absolute log2 fold change larger than 1 and an adjusted *P* value less than 0.01. Gene function analysis of these differentially expressed genes was performed using WebGestalt, and the *P* value was adjusted using the Benjamini−Hochberg method. The pathway was considered significantly enriched at an adjusted *P* value less than 0.05.

To further assess the enrichment of the functional pathways in each patient, we performed ssGSEA using the expression level data. All pathways were derived from the KEGG database and downloaded from the Molecular Signatures Database (MSigDB) (version 6.2). The enrichment score for each pathway in our sample was calculated by the R package GSVA [55]. A two-sided Student’s *t* test was used to compare the difference in the enrichment score between the two risk groups. The adjusted *P* value for each pathway was calculated using the Benjamini−Hochberg method. The pathway was considered significantly enriched at an adjusted *P* value less than 0.01.

### Analysis of differences in immune cell composition between the two risk groups

The cell fractions of 22 immune cell types in each patient were based on a deconvolution approach, CIBERSORT, and obtained directly from The Cancer Immunome Atlas (https://tcia.at/) (August 26, 2019). The difference in the relative composition of immune cell populations between the two risk groups was calculated by the Wilcoxon rank-sum test. *P* values were adjusted using the Benjamini−Hochberg method. Immune cell types with an adjusted *P* value less than 0.01 were deemed significant result.

## Supplementary Material

Supplementary Figure 1

Supplementary Tables
