# Supplementary material for: Risk stratification of cutaneous melanoma reveals carcinogen metabolism enrichment and immune inhibition in high-risk patients
Source: Aging (Albany NY). 2020 Aug 28;12(16):16457–75. doi: 10.18632/aging.103734 (PMC7485700; doi:10.18632/aging.103734)
Supplement: Supplementary Figure 1 [file aging-12-103734-s002..pdf]

## SUPPLEMENTARY FIGURE

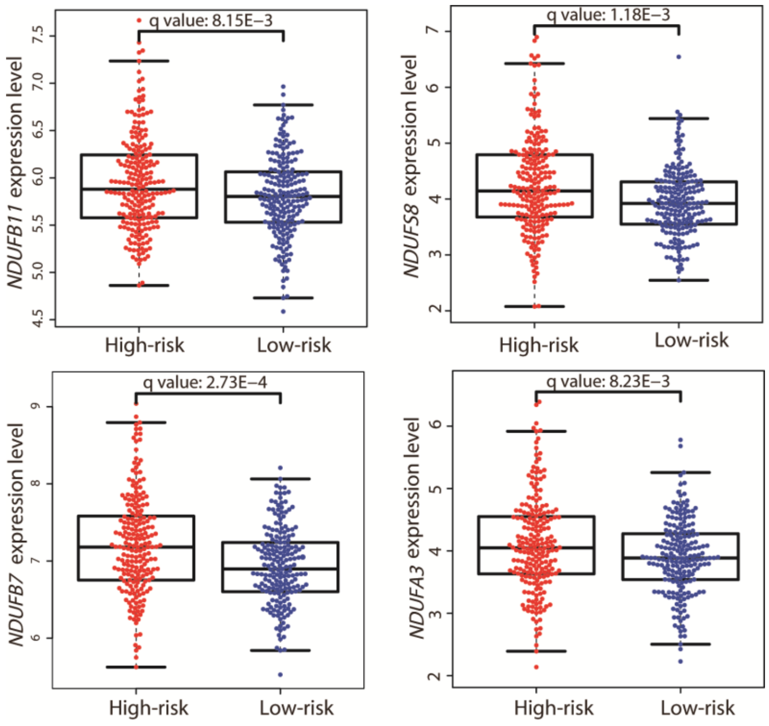


**Supplementary Figure 1. Expression differences in the four NADH dehydrogenase genes between the two risk groups.** Distribution of *NDUFA3*, *NDUFB7*, *NDUFS8*, and *NDUFB11* expression in the two risk groups. The expression levels are represented by log2-transformed RPKM values. The *P* value was calculated by a two-sided Student's *t* test and adjusted using the Benjamini–Hochberg method.
