# Supplementary material for: Risk stratification of cutaneous melanoma reveals carcinogen metabolism enrichment and immune inhibition in high-risk patients
Source: Aging (Albany NY). 2020 Aug 28;12(16):16457–75. doi: 10.18632/aging.103734 (PMC7485700; doi:10.18632/aging.103734)
Supplement: Supplementary Tables [file aging-12-103734-s001..pdf]

SUPPLEMENTARY TABLES

Supplementary Table 1. Information regarding the 21 genes following survival analysis.

Gene	Entrez ID	*P* value	coefficient	HR	95% CI
SATB1	6304	9.01E-5	-0.893	0.409	0.258-0.649
HN1L	90861	2.44E-4	0.928	2.53	1.514-4.229
CCL8	6355	6.58E-5	-0.939	0.391	0.243-0.631
TTC39C	125488	2.55E-4	-0.861	0.423	0.263-0.680
HPDL	84842	2.03E-4	0.875	2.398	1.491-3.858
OLFML2A	169611	8.75E-4	0.824	2.28	1.385-3.755
LMNTD2	256329	6.211E-3	-0.672	0.511	0.313-0.834
ATP11A	23250	3.173E-3	0.709	2.032	1.257-3.283
SLC5A3	6526	7.56E-6	-1.14	0.32	0.19-0.539
HEYL	26508	5.497E-3	0.677	1.968	1.209-3.201
BOK	666	2.01E-4	0.849	2.337	1.476-3.700
RBCK1	10616	1.056E-3	-0.792	0.453	0.278-0.736
CCT6B	10693	2.863E-3	-0.759	0.468	0.282-0.779
ABTB1	80325	5.107E-3	-0.643	0.526	0.333-0.831
CLEC18A	348174	5.438E-3	-0.612	0.542	0.35-0.841
MRPS6	64968	7.88E-4	-0.822	0.44	0.269-0.719
NXT2	55916	7.08E-5	-0.926	0.396	0.247-0.635
SPEF2	79925	2.208E-3	-0.708	0.492	0.310-0.782
KLK13	26085	6.35E-4	0.814	2.258	1.398-3.647
SPAG8	26206	9.86E-4	-0.765	0.465	0.292-0.742
COL22A1	169044	9.29E-4	-0.758	0.469	0.296-0.742

Supplementary Table 2. Risk prediction comparison with the validation cohorts.

GSE54467		Univariate		Multivariate	
**Variable**	**Group**	**HR (95% CI)**	***P* value**	**HR (95% CI)**	***P* value**
Risk score	High (n = 50)	1	**9.00E-5**		
	Low (n = 29)	0.257 (0.124-0.534)			
Age	≤ 60 (n = 47)	0.549 (0.309-0.975)	0.040		
	> 60 (n = 32)	1			
Gender	Male (n = 50)	1.090 (0.600-1.981)	0.800		
	Female (n = 29)	1			
Pathological stage	Stage I and II (n = 58)	1	0.100		
	Stage III (n = 20)	1.774 (0.860-3.658)			
**GSE65904**		**Univariate**		**Multivariate**	
**Variable**	**Group**	**HR (95% CI)**	***P* value**	**HR (95% CI)**	***P* value**
Risk score	High (n = 105)	1	**7.00E-5**	1	
	Low (n = 105)	0.453 (0.304-0.676)		0.385 (0.254-0.582)	**6.36E-6**
Gender	Male (n = 124)	1.335 (0.885-2.016)	0.200		
	Female (n = 86)	1			
Tumor stage	General (n = 23)	1	**1.00E-4**	1	
	In transit (n = 15)	0.736 (0.333-1.628)		0.591 (0.241-1.449)	0.250
	Local (n = 11)	0.316 (0.106-0.947)		0.232 (0.062-0.870)	0.030
	Primary (n = 15)	0.052 (0.007-0.394)		0.047 (0.004-0.563)	0.016
	Regional (n = 139)	0.354 (0.204-0.613)		0.315 (0.123-0.807)	0.016
Tissue	Cutaneous (n = 21)	1	**0.010**	1	
	Lymph node (n = 130)	2.884 (0.905-9.185)		1.117 (0.236-5.296)	0.889
	Subcutaneous (n = 33)	5.096 (1.513-17.160)		1.306 (0.285-5.985)	0.731
	Visceral (n = 10)	6.984 (1.743-27.987)		1.163 (0.209-6.471)	0.863
